# Identification of the long non-coding RNA *POU3F3* in plasma as a novel biomarker for diagnosis of esophageal squamous cell carcinoma

**DOI:** 10.1186/1476-4598-14-3

**Published:** 2015-01-21

**Authors:** Yu-Suo Tong, Xiao-Wei Wang, Xi-Lei Zhou, Zi-Hao Liu, Tong-Xin Yang, Wei-Hong Shi, Hai-Wei Xie, Jin Lv, Qing-Quan Wu, Xiu-Feng Cao

**Affiliations:** Department of Surgical Oncology, Nanjing Frist Hospital, Nanjing Medical University, Nanjing, Jiangsu China; Department of Medical Oncology, Huai’an First People’s Hospital Affiliated to Nanjing Medical University, Huai’an, Jiangsu China; Department of Radiation Oncology, Huai’an First People’s Hospital Affiliated to Nanjing Medical University, Huai’an, Jiangsu China; Department of Thoracic Surgery, Huai’an First People’s Hospital Affiliated to Nanjing Medical University, Huai’an, Jiangsu China

**Keywords:** Esophageal cancer, Long non-coding RNAs, Plasma, Biomarker, Diagnosis

## Abstract

**Background:**

Recent studies have demonstrated that long non-coding RNAs (lncRNAs) were present in the blood of cancer patients and have shown great potential as powerful and non-invasive tumor markers. However, little is known about the value of lncRNAs in the diagnosis of esophageal squamous cell carcinoma (ESCC). We hypothesized that ESCC-related lncRNAs might be released into the circulation during tumor initiation and could be utilized to detect and monitor ESCC.

**Methods:**

Ten lncRNAs (*HOTAIR*, *AFAP1-AS1*, *POU3F3*, *HNF1A-AS1*, *91H*, *PlncRNA1*, *SPRY4-IT1*, *ENST00000435885.1*, *XLOC_013104* and *ENST00000547963.1*) which previously found to be differently expressed in esophageal cancer were selected as candidate targets for subsequent circulating lncRNA assay. A four-stage exploratory study was conducted to test the hypothesis: (1) optimization of detected method to accurately and reproducibly measure ESCC-related lncRNAs in plasma and serum; (2) evaluation of the stability of circulating lncRNAs in human plasma or serum; (3) exploration the origin of ESCC-related lncRNAs in vitro and in vivo; (4) evaluation the diagnostic power of circulating lncRNAs for ESCC.

**Results:**

ESCC-related lncRNAs were detectable and stable in plasma of cancer patients, and derived largely from ESCC tumor cells. Furthermore, plasma levels of *POU3F3*, *HNF1A-AS1* and *SPRY4-IT1* were significantly higher in ESCC patients compared with normal controls. By receiver operating characteristic curve (ROC) analysis, among the three lncRNAs investigated, plasma *POU3F3* provided the highest diagnostic performance for detection of ESCC (the area under the ROC curve (AUC), 0.842; *p* < 0.001; sensitivity, 72.8%; specificity, 89.4%). Moreover, use of *POU3F3* and SCCA in combination could provide a more effective diagnosis performance (AUC, 0.926, *p* < 0.001, sensitivity, 85.7%; specificity, 81.4%). Most importantly, this combination was effective to detect ESCC at an early stage (80.8%).

**Conclusions:**

Plasma *POU3F3* could serve as a potential biomarker for diagnosis of ESCC, and the combination of *POU3F3* and SCCA was more efficient for ESCC detection, in particular for early tumor screening.

**Electronic supplementary material:**

The online version of this article (doi:10.1186/1476-4598-14-3) contains supplementary material, which is available to authorized users.

## Background

Esophageal squamous cell carcinoma (ESCC) is one of the most fatal malignancies in humans, causing more than 400000 deaths per year [1]. Patients trend to present with dysphagia at an advanced stage, and the 5-year survival rate is less than 15% [2]. Current biomarkers such as serum squamous cell carcinoma antigen (SCCA), carbohydrate antigen (CA) 19–9, and carcinoembryonic antigen (CEA) are the classic tumor markers commonly used in the management of patients with ESCC. However, these tumor markers, have limited utility in the early detection of ESCC due to lack of sufficiently high diagnostic sensitivity and specificity [3]. Although CYFRA 21–1 has been reported to have the higher sensitivity for diagnosing ESCC than the traditional tumor markers, the sensitivity is less than 10% for early detection of ESCC [4]. Therefore, the significance of exploration of new biomarkers with high sensitivity and specificity in early detection of ESCC should be emphasized.

Long non-coding RNAs (also known as lncRNAs), which are longer than 200 bases with lack of protein-coding capability, play critical roles in tumor initiation, progression and metastasis by modulating oncogenic and tumor-suppressing pathways [5, 6]. Previous studies have proved that lncRNAs are frequently dysregulated expression in different kinds of tumors, including ESCC [7]. The aberrant expression of lncRNAs have been reported to serve as potential diagnostic or prognostic biomarkers for many human malignancies such as breast, lung, liver, and colon cancers [8–11]. Although these lncRNAs have shown great promise as a new kind of tumor markers, they cannot be used for clinical screening purposes because of difficulty in getting biopsies of tissue from patients suspected to be ESCC.

Circulating RNA in plasma or serum has been an emerging field for noninvasive diagnostic applications [12]. More recently, MicroRNAs (miRNAs) have been detected in human peripheral blood, being remarkably stable in spite of the high amounts of endogenous ribonuclease in the blood of cancer patients [13]. Moreover, several clinical trials have been approved by the FDA to assess the value of serum miRNAs in cancer diagnosis (http://clinicaltrials.gov) [14]. At present, several lncRNAs have been characterized as potential tumor markers in human fluids. For example, *MALAT1* was found to be significantly up-regulated in plasma of prostate cancer patients, and could be used to discriminate cancer patients from healthy controls [6, 15]. In gastric cancer patients, plasma *AA174084* levels dropped markedly on day 15 after surgery compared with preoperative levels and were associated with invasion and lymphatic metastasis [6, 15]. However, to our knowledge to date, no study has been performed regarding the circulating lncRNAs for early detection of ESCC patients.

In the present study, 10 lncRNAs *(HOTAIR, AFAP1-AS1, POU3F3, HNF1A-AS1, 91H, PlncRNA1, SPRY4-IT1, ENST00000435885.1, XLOC_013104 and ENST00000547963.1*) that were previously reported with deregulated expression in esophageal cancer were selected as candidate diagnostic makers [16–23]. They were examined in tissues and plasma, and their potential use as tumor markers for ESCC detection were evaluated. We hypothesized that these ESCC-related lncRNAs might be released into the circulation during ESCC initiation and could be utilized to detect and monitor ESCC. To test the hypothesis, the following crucial questions need to be addressed: 1) the stability of circulating lncRNAs in plasma and serum, 2) the relationship between tumor tissue lncRNAs and circulating lncRNAs, 3) and the source of circulating lncRNAs (from cancer cells or from normal blood cells).

## Results

### Evaluation and screening of candidate endogenous control

In order to evaluate and screen the optimal endogenous control for lncRNAs analysis of tissues and cells, the candidate reference genes (*GAPDH, TBP, β-actin* and *HPRT1*) were measured in 21-pair ESCC tumor tissues and adjacent normal tissues. The raw Ct values were provided in the supporting information (Additional file 1: Table S1). Finally, through four common algorithms (Normfinder, geNorm, BestKeeper, and the Comparative △△Ct method) which were described elsewhere in details [24], *GAPDH* was identified as the most stable endogenous control for tissues lncRNAs analysis (Table 1).

**Table 1:** **Results of using RefFinder to evaluate and screen the most stable endogenous control for tissues lncRNAs analysis**

Method	Ranking order (better–good–average)			
△△Ct method	*GAPDH*	*HPRT1*	*β-actin*	*TBP*
BestKeeper	*GAPDH*	*β-actin*	*HPRT1*	*TBP*
Normfinder	*GAPDH*	*β-actin*	*HPRT1*	*TBP*
GeNorm	*GAPDH*	*HPRT1*	*β-actin*	*TBP*
Recommended comprehensive ranking	*GAPDH*	*HPRT1*	*β-actin*	*TBP*

However, for the early stage of circulating lncRNAs study, no consensus exists on the use of endogenous control for detection the lncRNAs in plasma. *GAPDH*, which was reported to be stably expressed in plasma and was selected as internal control in plasma by others before [15], was regarded as an ideal internal control for plasma assay. Therefore, *GAPDH* expression was measured in 270 plasma samples (147cases and 123controls). Results revealed that *GAPDH* level was stable in human plasma and was not affected by age, sex, and pathology (Additional file 2: Table S2).

### Selection and detection of ESCC-related lncRNAs

**Figure 1:**
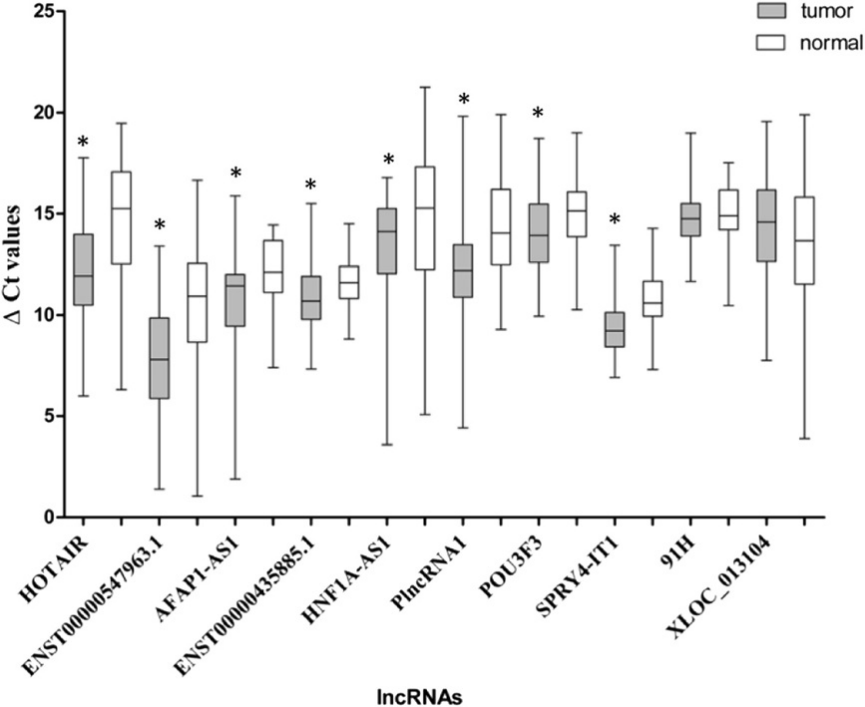
**Validation of ESCC-related lncRNAs expression in ESCC tissues.** △Ct method was used to calculate lncRNA expression, which was normalized to *GAPDH*, and smaller ΔCt value indicated higher expression. Horizontal lines inside the box plots represent the median, boxes represent the interquartile range, and error bars represent 97.5th and 2.5th percentiles. *P < 0.05.

On the basis of previous study, 10 lncRNAs (*HOTAIR, AFAP1-AS1, POU3F3, HNF1A-AS1, PlncRNA1, SPRY4-IT1, ENST00000435885.1, ENST00000547963.1, 91H and XLOC_013104*) which have been reported to be differently expressed in esophageal cancer were selected in the present study. All the lncRNAs were then subjected to qPCR validation, which was performed in 48 pairs of ESCC tumor tissues and adjacent normal tissues. Among them, *HOTAIR, AFAP1-AS1, POU3F3, HNF1A-AS1, PlncRNA1, SPRY4-IT1, ENST00000435885.1* and *ENST00000547963.1* were significantly higher in most of ESCC tumor tissues compared with paired adjacent normal tissues (Figure 1). However, expression of *91H* and *XLOC_013104* did not show any significantly different expression between the two groups and therefore were ruled out in the subsequent study (Figure 1).

### The presence of ESCC-related lncRNAs in human plasma

The detection of lncRNAs from plasma or serum with sufficient sensitivity is a prerequisite of developing circulating lncRNAs-based tumor markers. Therefore, optimization of extraction method was very important (detailed in Additional file 3: Text S1).

**Figure 2:**
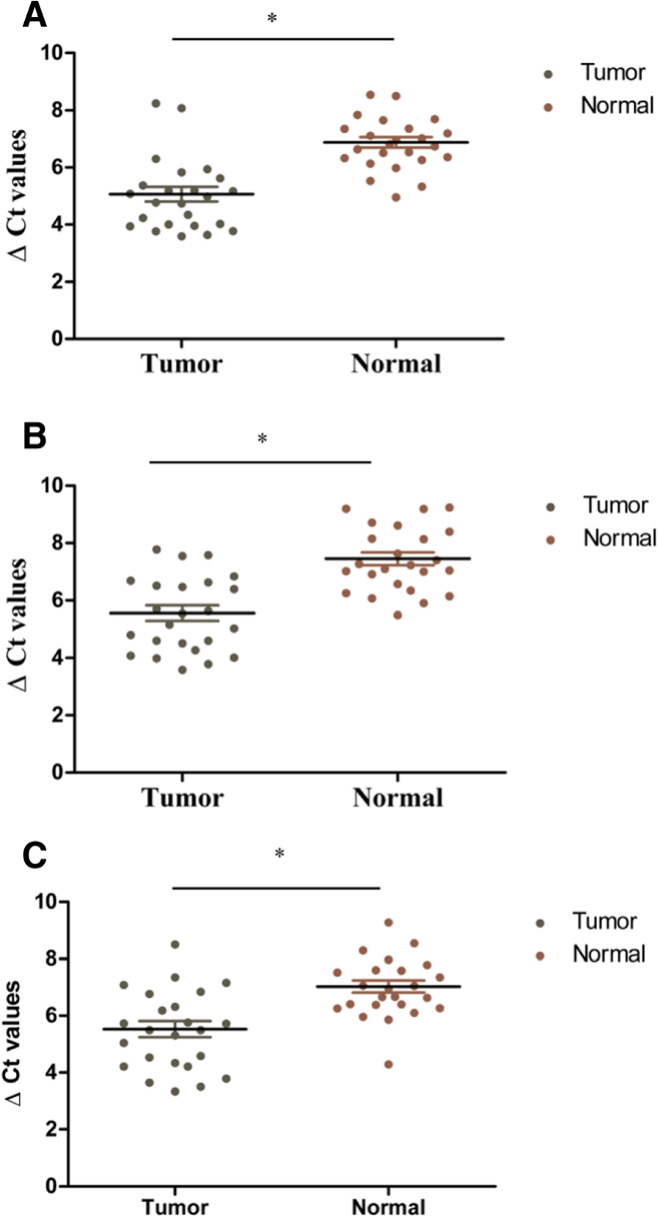
**ESCC-related lncRNAs were detectable in plasma of tumor patients and normal control. (A-C)** Plasma levels of *POU3F3* **(A)**, *HNF1A-AS1* **(B)**, and *SPRY4-IT1* **(C)** were measured in 24 clinical samples and 24 cancer free controls. Horizontal bars indicate median and interquartile range. Circulating lncRNAs expressions were calculated using △Ct method. Statistical differences were analyzed using Mann–Whitney test. *p < 0.001.

To explore whether these ESCC-related lncRNAs could reach the circulation at levels sufficient to be detectable, qPCR was then used to examine expression of selected 8 lncRNAs in 48 plasma samples (24 clinical samples and 24 normal controls). Of these lncRNAs, as shown in Figure 2A-C, the levels of *POU3F3* (*p* < 0.001), *HNF1A-AS1* (*p* < 0.001) and *SPRY4-IT1* (*p* < 0.001) were significantly higher in ESCC patients comparing with normal controls. Moreover, to make sure whether these lncRNAs were present in plasma, the qPCR products were further validated by using traditional Sanger-based method. As expected, their sequences were identical to those derived from *POU3F3, HNF1A-AS1* and *SPRY4-IT1* (Additional file 4: Figure S1). The results indicated that ESCC-related lncRNAs could enter into the circulation and their differentiate expression in plasma could be used as diagnostic markers for ESCC. However, *PlncRNA1, HOTAIR* and *ENST00000547963.1* had a detection rate of less than 60% in both ESCC plasma samples and normal controls, and therefore were excluded. In addition, *ENST00000435885* (*p* = 0.386) and *AFAP1-AS1* (*p* = 0.232) were also excluded because there were no significant differences in their expressions between the two groups (Additional file 5: Figure S2).

### Stability of ESCC-related lncRNAs in human plasma

**Figure 3:**
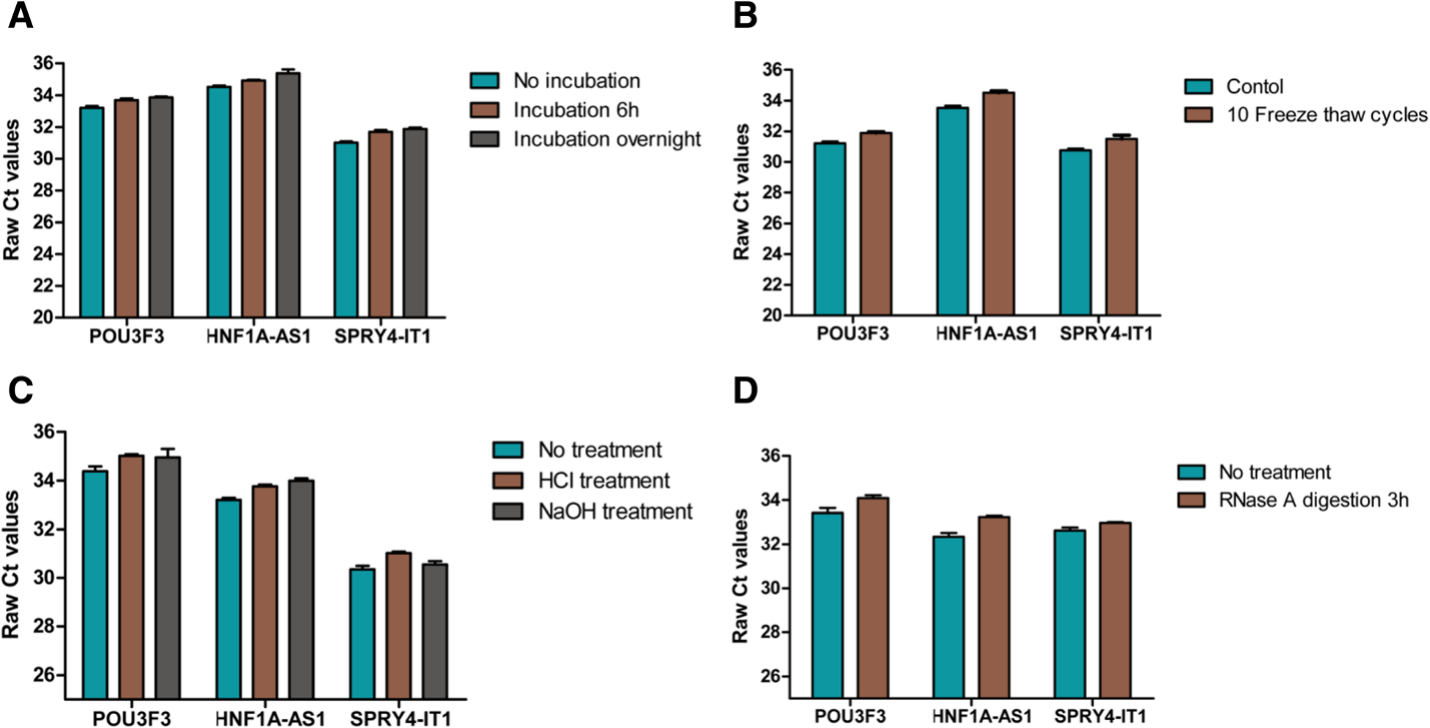
**Stability of plasma ESCC-related lncRNAs.** *POU3F3*, *HNF1A-AS1*, and *SPRY4-IT1* shown no significant degradation when plasma was treated with prolonged room temperature incubation time **(A)**, or multiple freeze-thaw cycles **(B)**, or low (pH = 1) or high (pH = 13) pH solution **(C)**, or RNase A digestion **(D)**. Data presented as raw Ct value, and each bars represented the mean (SD) (n = 3).

To determine the stability of lncRNAs in plasma, given that this is a crucial prerequisite for utility as tumor markers, plasma samples were left under harsh conditions including incubation at room temperature for 0, 6, and 24 h, repeated freeze-thaw cycles, or low/high pH, respectively. A total of 12 ESCC plasma samples were assessed, and they were divided into three portions. At room temperature, the levels of *POU3F3, HNF1A-AS1 and SPRY4-IT1* were not significantly altered from 0 h to 6 h, but then slightly decreased at 24 h when compared with 6 h (Figure 3A). The stability of plasma lncRNAs was treated with strong acid, strong base, or 10 multiple freeze-thaw cycles, respectively, for further validation. As shown in Figure 3B and C, lncRNAs in treated plasma remained stable compared to plasma not treated Given that circulating lncRNA has been thought to be unstable, because of the existence of the endogenous RNase in blood. Three additional ESCC plasma samples were incubated with RNase A for 3 h at room temperature to determine whether lncRNAs could be degradated. Results indicated that RNase A had hardly any effect on plasma level of *POU3F3, HNF1A-AS1 and SPRY4-IT1* (Figure 3D).

### Comparison of ESCC-related lncRNAs between plasma and serum

**Figure 4:**
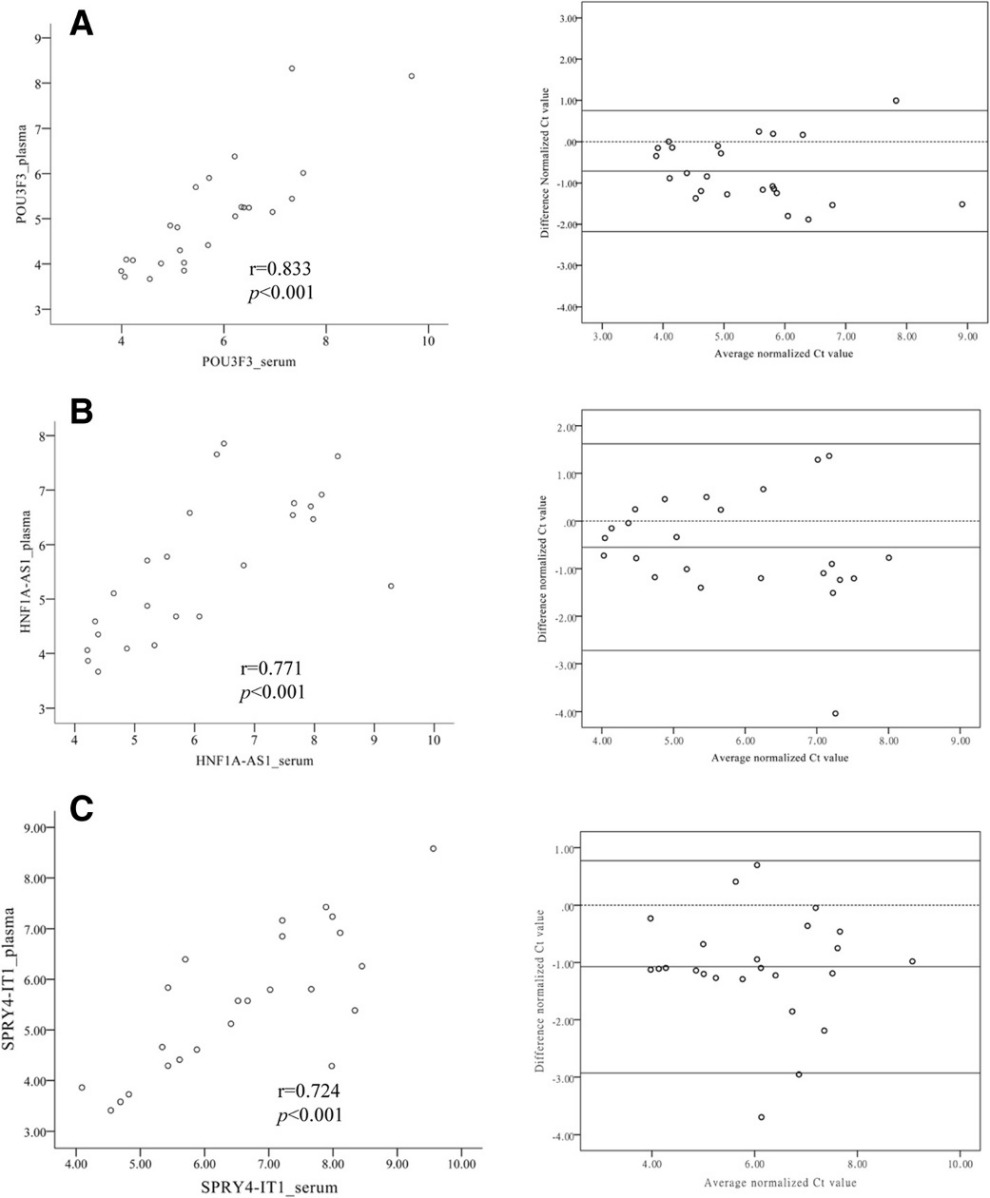
**Correlation of ESCC-related lncRNAs expression between plasma and serum.** Left panel, linear correlation plot of POU3F3 **(A)**, HNF1A-AS1 **(B)** and SPRY4-IT1 **(C)** level from plasma (y-axis) versus from serum (x-axis). There was a high correlation comparing the indicated lncRNAs levels between plasma and serum. Spearman’s rank analysis was used to identify the correlation of lncRNAs levels between plasma and serum. Right panel, Bland-Altman plot of the difference between plasma and serum ESCC-related lncRNAs level (y-axis) versus their average (x-axis). Horizontal solid lines in the middle represent the mean difference. Upper and lower solid lines represent the limits of agreement (95% confidence intervals).

To test whether there was a relationship between plasma and serum lncRNAs level, *POU3F3*, *HNF1A-AS1* and *SPRY4-IT1* were measured in EDTA-plasma samples and clotted blood from the same individuals at the same blood draw. As shown in Figure 4, measurements obtained from plasma and serum were strongly correlated for *POU3F3* (*r* = 0.833, mean differences = -0.71 ± 0.75, Figure 4A), *HNF1A-AS1* (*r* = 0.771, mean differences = -0.55 ± 1.12, Figure 4B) and *SPRY4-IT1* (*r* = 0.724, mean differences = -1.07 ± 0.95, Figure 4C), respectively. The results suggested that serum and plasma samples were both acceptable for evaluation of lncRNAs as blood-based tumor markers.

### Origin of ESCC-related lncRNAs in plasma

To further test the hypothesis that plasma lncRNAs were primarily released or leaked from the tumor cells. Five independent experiments were carried out to determine the source of ESCC-related lncRNAs in plasma.

**Figure 5:**
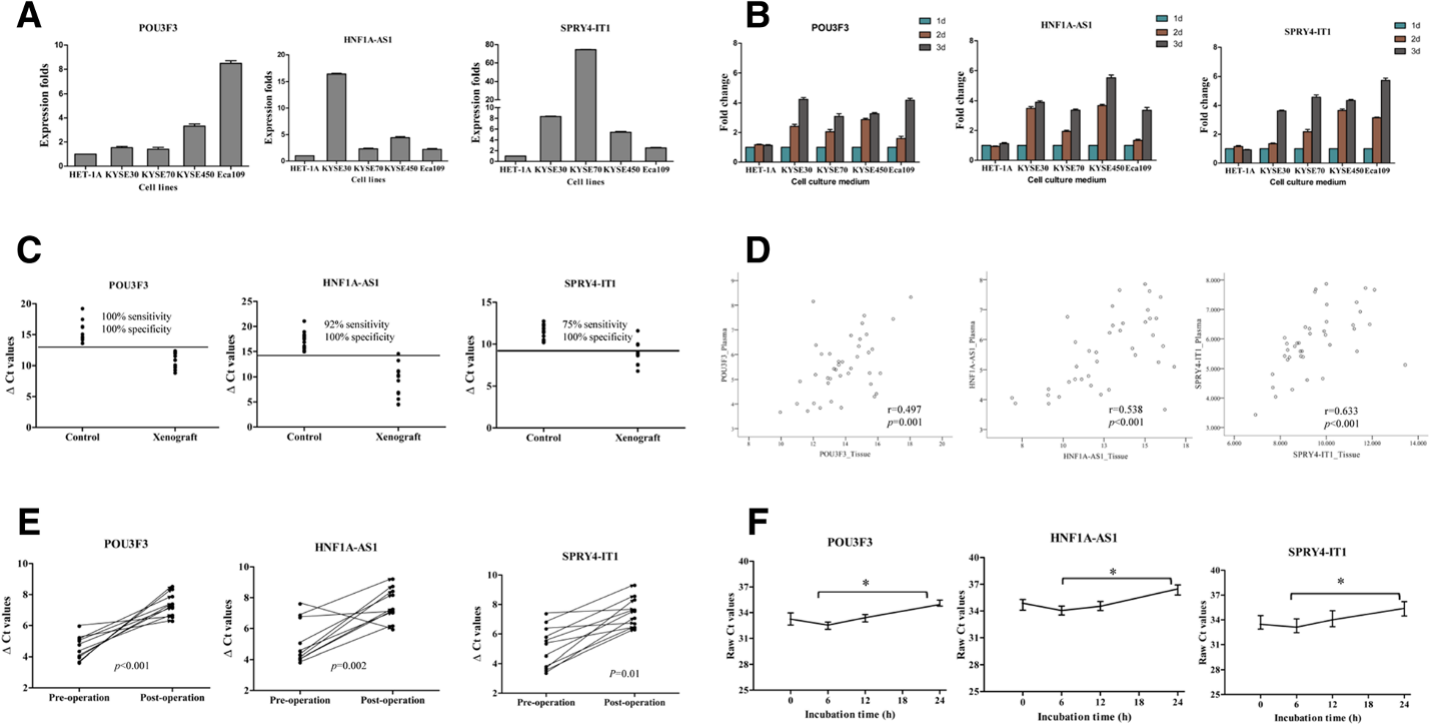
**Origin of circulating lncRNAs. (A)** Quantitative real time polymerase chain reaction (qPCR) was used to measure ESCC-related lncRNAs expression in five esophageal cells. *GAPDH* was used as a normalization control. **(B)** ESCC-related lncRNAs could be secreted into the cell culture medium. Data presented as relative lncRNAs fold change. **(C)** ESCC-related lncRNAs could enter into the circulation of xenograft-bearing mice. **(D)** Spearman’s rank correlation scatter plot of ESCC-related lncRNAs levels in tumor samples and plasma. Data were presented as △Ct values normalized to *GAPDH*. (E) ESCC-related lncRNAs expressions were significantly declined in post-operative samples compared with that in pre-operative samples. **(F)** Effect of delayed blood centrifugation on plasma ESCC-related lncRNAs expressions. At room temperature, ESCC-related lncRNAs levels of unfiltered plasma was slightly increased from 0 h to 6 h, but then significantly decreased at 24 h when compared to 6 h. *p < 0.05.

In the first experiment, qPCR was used to measure the *POU3F3*, *HNF1A-AS1* and *SPRY4-IT1* expression in different five esophageal cell lines (including four ESCC cells: KYSE30, KYSE70, KYSE450, and Eca 109, and one normal human esophageal epithelial cell line: HET-1A), and each cell culture medium which was incubated for 1, 2, and 3d (Figure 5A and B). Data presented in Figure 5B indicated that ESCC-related lncRNAs could enter into the cell culture medium at a detectable level and were steadily increased among the three incubation time points in the all four ESCC cell lines; however, no significant changes were observed in normal esophageal epithelial cell culture medium.

In the second experiment, a mouse ESCC xenograft model system was established to further investigate whether ESCC-related lncRNAs could enter the circulation at a measurable level. Blood samples were collected 4 weeks after implanting the KYSE30 cells or PBS, and the blood processing and RNA isolation were the same as those described for human plasma. Then the qPCR results demonstrated that the presence of tumor was the major contributor to plasma lncRNAs (Figure 5C).

In the third experiment, qPCR was used to measure the ESCC-related lncRNAs expression in 24 ESCC tumor tissues and paired plasma samples, and then correlation of the levels of ESCC-related lncRNAs between the two groups was analyzed. As shown in Figure 5D, a moderate significant correlation was observed for *POU3F3* (*r* = 0.497, *p* = 0.001), *HNF1A-AS1* (*r* = 0.538, *p* < 0.001), and *SPRY4-IT1* ((*r* = 0.633, *p* < 0.001), respectively, which was consistent with our previous hypothesis.

Since circulating lncRNAs were primarily released or leaked from tumor cells, they would presumably revert to normal after the tumor has been resected. To test this hypothesis, the fourth experiment was carried out to investigate the differences in ESCC-related lncRNAs in plasma before and 14d after surgery. As expected, plasma levels of *POU3F3* (*p* < 0.001, Wilcoxon test) *HNF1A-AS1* (*p* = 0.002, Wilcoxon test) and *SPRY4-IT1* (*p* = 0.001, Wilcoxon test) were markedly declined 14d after surgical treatment compared with before surgery (Figure 5E).

In addition, previous studies reported that some circulating RNAs could derive from the blood cells [25]. Therefore, the fifth experiment with delayed blood centrifugation after venesection was performed to further clarify the origin of circulating ESCC-related lncRNAs. A total of 12 EDTA-blood samples collected from healthy participants were used in this part of study. Four blood samples were incubated at room temperature for 0, 6, 12, and 24 h, and the second 4 blood samples were left at 4°C for 0, 6, 12, and 24 h. The last 4 blood samples were treated as the same as the first part, but the collected plasma was filtered through a 0.22 μm filter. After the treatment, plasma was then collected for RNA isolation. The results showed that, at room temperature, *POU3F3*, *HNF1A-AS1* and *SPRY4-IT1* expression in the unfiltered plasma was slightly increased at 6 h compared to 0 h, but then significantly declined at 24 h compared to 6 h (Figure 5F). However, no significant differences were observed when the EDTA-blood was left at 4°C or for the filtered plasma (Additional file 6: Figure S3).

### Correlation between plasma lncRNAs and clinicopathological characteristics

To further determine whether the three lncRNAs levels in ESCC plasma were associated with specific clinicopathologic parameters, such as gender, tumor size, histologic grade, smoking status, alcohol consumption, TNM stage, and clinical stage. *POU3F3*, *HNF1A-AS1* and *SPRY4-IT1* expression levels were measured in all 147 ESCC patients; however, there was no significant association between plasma lncRNAs and clinicopathological parameters (Additional file 7: Table S3).

### Evaluation of lncRNAs in plasma as novel tumor markers for ESCC

**Figure 6:**
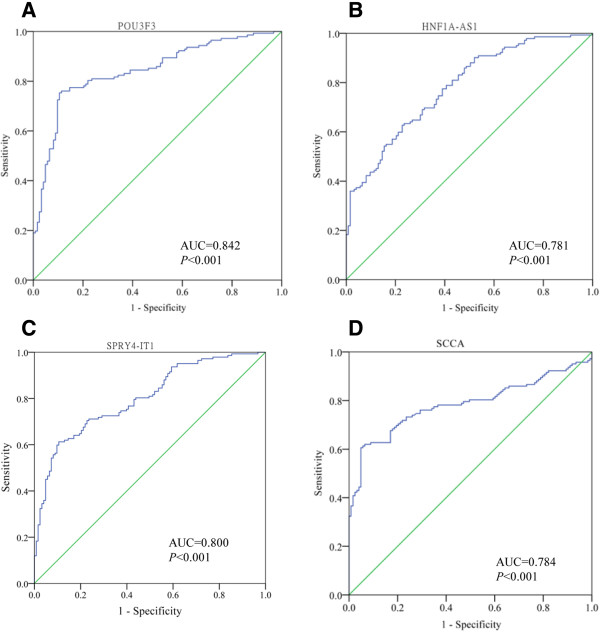
**Evaluation of plasma lncRNAs for detection of ESCC.** Receiver operating characteristics (ROC) curves were drawn with the data of plasma lncRNAs from 147 patients with ESCC and 123 healthy controls. **(A-D)** ROC-AUC for detecting ESCC from healthy controls (*POU3F3*, 0.842, *p* < 0.001; *HNF1A-AS1*, 0.781, *p* < 0.001; *SPRY4-IT1*, 0.800, *p* < 0.001; SCC*A*, 0.784, *p* < 0.001).

To investigate the characteristics of the three ESCC-related lncRNAs as potential tumor markers of ESCC, Receiver operating characteristics (ROC) curves and the area under the ROC curves (AUC) were performed on data from all subjects, including 147 ESCC patients and 123 healthy donors. The ROC curves illustrated strong separation between the ESCC patients and control group, with an AUC of 0.842 (95% CI: 0.794 – 0.890; *p* < 0.001) for *POU3F3*, 0.781 (95% CI: 0.727 – 0.835: *p* < 0.001) for *HNF1A-AS1*, and 0.800 (95% CI: 0.748 – 0.853: *p* < 0.001) for *SPRY4-IT1*, respectively, compared with classic tumor marker SCCA (ng/ml) with an AUC of 0.784 (95% CI: 0.727 – 0.841; *p* < 0.001) (Figure 6). Moreover, plasma level of *POU3F3* could discriminate ESCC from normal controls with 72.8% sensitivity and 89.4% specificity, although the levels of *HNF1A-AS1* and *SPRY4-IT1* in plasma were less sensitive (32.7% and 48.2%) for ESCC detection. Therefore, of the three lncRNAs in this analysis, *POU3F3* provided the highest diagnostic power for the detection of ESCC, suggesting that plasma *POU3F3* could serve as a promising tumor marker for ESCC diagnosis.

### Combination of *POU3F3*and SCCA for ESCC diagnosis

**Figure 7:**
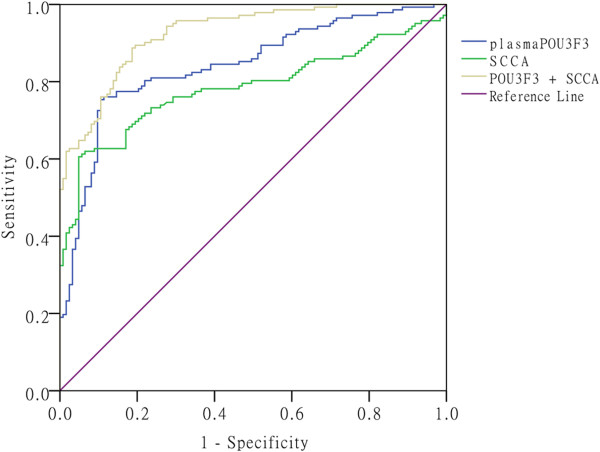
**ROC corves to compare the diagnostic performance of SCCA,** ***POU3F3*** **, and a combination of SCCA and** ***POU3F3*** **, to discriminate ESCC from normal controls.** The combination of SCCA and *POU3F3* provided a more powerful differential diagnosis compared with SCCA and *POU3F3* alone (SCCA+ *POU3F3*, 0.926, *p* < 0.001; *POU3F3*, 0.842, *p* < 0.001; SCCA, 0.784, *p* < 0.001).

There is increasing evidence showing that combination several tumor markers could improve diagnostic accuracy. In this study, we determined whether the combination of *POU3F3* and SCCA would provide a more effective diagnosis performance for detection of ESCC. As shown in Figure 7, by binary logistic regression, combination of *POU3F3* and SCCA yielded an AUC of 0.926 (95% CI: 0.896 – 0.955; *p* < 0.001), which was significantly improved compared with SCCA (AUC = 0.784) or lncRNA *POU3F3* (AUC = 0.842) alone. The sensitivity, specificity, accuracy of SCCA, *POU3F3,* and the combination (SCCA + *POU3F3*) for distinguishing ESCC from healthy controls were summarized in Table 2.

**Table 2:** **Performance of SCCA,** ***POU3F3*** **, and both SCCA and POU3F3 in the differential diagnosis ESCC from healthy participants**

	Sensitivity	Specificity	Accuracy	Youden index	True positive	True negative	False-positive	False-negative
SCCA	59.2%	93.5%	74.8%	52.7%	87	115	8	60
*POU3F3*	72.8%	89.4%	80.3%	62.2%	107	110	13	40
SCCA + *POU3F3*	85.7%	81.4%	83.7%	67.1%	126	100	23	21

*Abbreviations:* SCCA squamous cell carcinoma antigen.

### Tumor stage and performance of the combination of *POU3F3*and SCCA

The key aim of this work was to diagnose ESCC patients at as early stage as possible at the time cancer therapy could be more likely successful. Therefore, the diagnostic positivity rate between different clinical stages was then investigated. As shown in Table 3, in stage I, the diagnostic positivity rate for SCCA, *POU3F3*, and both in combination was 26%, 69.2%, and 80.8%, respectively.

**Table 3:** **Performance of SCCA,** ***POU3F3*** **and both SCCA and** ***POU3F3*** **in the detection of different clinical stage ESCC**

	Clinical stage					***P*** (Pearson Chi-square)
	I	II	III	IV	Total	
SCCA	26% (7/26)	35.9% (14/39)	76.4% (39/51)	87.1% (27/31)	59.2% (87/147)	0.000
*POU3F3*	69.2% (18/26)	66.6% (26/39)	78.4% (40/51)	74.2% (23/31)	72.8% (107/147)	0.455
SCCA + *POU3F3*	80.8% (21/26)	76.9% (30/39)	90.2% (46/51)	93.5% (29/31)	85.7% (126/147)	0.147
*P* (combination vs. SCCA)	0.000	0.001	0.109	0.671	0.000	

*Abbreviations:* SCCA squamous cell carcinoma antigen, statistic analysis, Pearson Chi-Square or Fisher’s Exact Test.

## Discussion

Recently, it has been demonstrated that the cell-free nucleic acids are detectable in plasma and serum of cancer patients and therefore may be utilized as a tool for cancer diagnosis [26]. Although numerous studies have focused on miRNAs as potential tumor markers for cancer diagnosis and prognosis prediction, the diagnostic utility of plasma lncRNAs in ESCC has never been studied.

In the present study, the initial ESCC-related lncRNAs screening was performed based on different expression profiling between ESCC tumor samples and matched normal samples that have been demonstrated in previous studies. All lncRNAs of interest were then subjected to qPCR validation. Eight lncRNAs were identified and then further measured their expression levels in plasma from ESCC patients and healthy subjects. The results demonstrated that the levels of *POU3F3, HNF1A-AS1* and *SPRY4-IT1* were significantly higher in plasma from ESCC patients compared with normal controls, providing strong evidence that ESCC-related lncRNAs could be released into the circulation and that their different expression profiles in plasma could be used as diagnostic markers for ESCC. Among the three lncRNAs, *POU3F3* provided the highest diagnostic power for detection of ESCC (AUC = 0.842; sensitivity, 72.8%; and specificity, 89.4%), suggesting that plasma *POU3F3* could serve as a promising tumor marker for ESCC detection. Furthermore, use of *POU3F3* and SCCA in combination could provide a more powerful differential diagnosis between ESCC patients and healthy controls than use of *POU3F3* or SCCA alone. Therefore, both in combination could be used as a diagnostic tool for screening apparently healthy individuals. Most importantly, the results indicated that plasma *POU3F3* was more effective than SCCA for early detection of ESCC (69.2% vs 26%), and that the positivity rate of both in combination was significantly improved compared with SCCA or *POU3F3* alone. In addition, we also proved the mirVana PARIS Kit (Ambion 1556, USA) approach as the most effective RNA extraction method, and both plasma and serum would be acceptable for evaluation of lncRNAs as blood-based tumor markers. This is the first time to systematically characterize circulating lncRNAs in plasma as diagnostic markers for ESCC.

Recently, many tumor markers such as SCCA, CEA, CA19-9, MMP-9, IL-6, CYFRA 21–1, DKK-1, M-CSF, *MiR-18a*, *MiR-1246* and many other genes were evaluated for ESCC diagnosis [3, 27–30]. However, there was no sufficient sensitivity and specificity for these biomarkers, even for the most commonly used biomarkers such as serum CEA and SCCA*.* Mealy K reported that the individual sensitivities of CEA and SCCA for the diagnosis of ESCC were about 28% and 32%, respectively [28]. Yamamoto K study demonstrated that the sensitivity of CYFRA 21–1 was only 47.9%, although the specificity was 100% [31]. MMP-9 has been shown to have higher diagnostic sensitivity of ESCC compared to SCCA, but its diagnostic performance was poorer [31]. Serum *MiR-1246* improved the discriminatory power, but sensitivity and specificity were lower [30, 32]. This study was sought to find novel markers that could improve early detection of ESCC. Our results demonstrated that plasma lncRNA *POU3F3* was a promising tumor marker, which effectively supplemented the serum SCCA for ESCC detection.

In the past several years, qPCR has been considered to be a reliable method for quantitative gene expression due to its accuracy, sensitivity, specificity, reproducibility and robustness [33]. However, to produce accurate results in qPCR assays the use of robust normalization strategy is important [34]. Generally, the use of reference gene as endogenous controls is the most commonly method for normalizing qPCR gene expression data. Currently, several mathematical algorithms which are specifically developed for reference gene evaluation and selection deliver suitable reference genes with the lowest variation and with high stability across the biological samples. The four commonly used approaches are: (1) NormFinder algorithm which is a statistical model that estimates the overall variation of gene expression for each candidate reference gene and delivers a stability value. (2) GeNorm. (3) BestKeeper, a Microsof Excel-based tool, uses pair-wise correlation and (4) the comparative delta Ct [35]. Therefore, in this study we paid special attention on reference genes selection. All candidate reference genes for ESCC tissue lncRNAs normalization were chosen based on their stably expressed in different human tissues or were once used in ESCC qPCR study. Finally through mathematical algorithms, *GAPDH* was selected for normalization of tissue lncRNAs measurement because of its best reference performance (Table 1). This was in agreement with Li W [18], using *GAPDH* normalization lncRNA *POU3F3* expression in tissue samples of ESCC patients. As there was no established endogenous control for detection of plasma or serum lncRNAs, *GAPDH* was selected as a potential reference gene. And we observed that plasma *GAPDH* expression level was stable between the ESCC group and normal control group and was not affected by age, sex, and pathology. However, in the previous study by Ng EK et al. [36]. *GAPDH* seemed to be no feasible reference gene because of its expression level in HCC patients was significantly higher than those in health individuals. One possible explanation was that different diseases and selected primer pair was not the best choice for measuring gene expression could influence the expression of *GAPDH*.

Circulating lncRNAs were thought to be unstable because of the high level of RNase activity in plasma, and in cancer patients, increased plasma RNase has been detected [12]. In the present study, we confirmed that circulating lncRNAs were remarkably stable even when treated directly with RNase A digestion. These findings were consistent with those in patients with prostate cancer [6]. However, the precise mechanism used to explain why circulating lncRNAs are resistant to endogenous RNase digestion remains largely unknown. One explanation is that they are packaged in some kinds of microparticles, such as exosomes, microvesicles, apoptotic bodies, and apoptotic microparticles [14, 37]. Our results of the blood-processing assay support this explanation. Recently, it has been hypothesized that circulating RNAs could be modified in certain ways, including methylation, adenylation, and uridylation which make them resistant to RNase digestion [38]. Another possible explanation is that lncRNAs often forms secondary structures, and relatively more stable, which could facilitate their detection as free nucleic acids in body fluids such as blood and urine [39]. The mechanism of resistance of circulating lncRNAs to RNase deserves further study. In the present study, we also demonstrated that plasma lncRNAs were resistant to multiple freeze-thaw cycles, strong acid and base treatment. However, when plasma or unprocessed blood was subjected to extended room temperature incubation, the concentrations of lncRNAs were even more variable. Such artifactual fluctuations in lncRNAs concentrations may be attributable to two factors: the secreted lncRNA from necrotic and/or apoptotic blood cells; the stability of original and the newly secreted lncRNA [12]. Taking the unprocessed EDTA-blood for instance (Figure 5F); an initial increase in lncRNAs concentrations at 6 h may be the result of the newly released lncRNAs from blood cells. After that, the lncRNA concentrations started to decline which may be attributable to the degradation of the newly secreted lncRNAs. Based on the above results, we recommend that the unprocessed EDTA-blood should be stored at 4°C.

The release of nucleic acids into the blood is thought to be associated with apoptosis and necrosis tumor cells from the tumor microenvironments and is also the results of secretion [40, 41]. In the previous study of circulating lncRNA *LIPCAR* in heart failure patients from Kumarswamy and his colleges, who suggested that a good proportion of mitochondrial lncRNAs detected in circulation might come from the heart [42]. On the contrary, Prichard et al. provided the pioneering evidence that blood cells were the major contributor to the circulating miRNAs and that perturbations in blood cell counts and hemolysis could alter plasma miRNAs levels by up to 50-fold [25]. Another possible origin may be that the circulating miRNAs of cancer patients were not just a result of cancer but may actively contribute to cancer defense, because the population of circulating miRNAs correlated tightly with the immune response [40]. Currently, there were three major hypotheses for circulating miRNAs to enter into the circulation: energy-free passive leakage of cellular miRNAs into circulation; active and selective secretion of miRNAs in response to various stimuli as microvesicle-free miRNAs; and active secretion via cell-derived microvesicles [38]. These theories could also be utilized to explain the origin of circulating lncRNAs. In this study, we found that ESCC-related lncRNAs could enter into the cell culture medium at a measurable level. Furthermore, ESCC-related lncRNAs were derived from tumor cells as evidenced with the xenograft assay, the relationship of ESCC-related lncRNAs levels between the tumor samples and plasma matched for the same individuals, and the changes of plasma ESCC-related lncRNAs concentrations between pre-Op and post-Op. However, the delayed blood processing assay suggested that the circulating lncRNAs could also be released from blood cells although their contribution was minor. Therefore, to avoid the origin of lncRNAs from blood cells, the unprocessed blood should be stored at 4°C and further processed as soon as possible. In addition, the stable levels of the three lncRNAs in the filtered plasma could be used to explain why increased plasma lncRNAs detected in unfiltered blood samples were largely from microparticles. Some patients, however, exhibited a different pattern of lncRNAs expression, low expression in plasma with high levels in ESCC tissues. At present, it was difficult to explain such phenomena; one possible hypothesis for that phenomenon could be the heterogeneity of primary tumor. At present, the exact biological roles of circulating lncRNAs in cancer patients remain unclear. Are they merely molecular remnants of necrotic tumor cells or do they play important roles in cell-to-cell communication? Obviously, more investigations will be needed to solve the exciting questions.

### Limitation of the study

However, research limitations exist in our study, such as modest sample size, relatively low sensitivity qPCR method and failure in deep functional investigation. Further studies focused on the biological role of plasma lncRNAs are needed.

## Conclusions

In summary, our results suggested that increased plasma lncRNA *POU3F3* can be used as ideal tumor biomarker for ESCC detection, and that combination of *POU3F3* and SCCA has a higher positive diagnostic rate of ESCC than *POU3F3* or SCCA alone, in particular at its early stage.

## Material and methods

### Ethical approval

Written informed consent was obtained from each participant prior to blood and tumor samples collection. All of the clinical samples were obtained from Nanjing Medical University Nanjing First Hospital (Nanjing, China) and Huai’an First Hospital (Huai’an, China). The study protocol was approved by the Clinical Research Ethics Committee of Nanjing First Hospital and Huai’an First Hospital, respectively.

### Clinical samples and plasma preparation

In this study, 147 consecutive hospitalized patients who had newly diagnosed with ESCC were selected from Nanjing Medical University affiliated Nanjing Hospital (n = 53) and Nanjing Medical University affiliated Huai’an First Hospital (n = 94) between January 2013 and May 2014. All patients selected met the following inclusion criteria: pathological examination confirmed primary ESCC by available biopsy samples; and no anticancer treatments were given before surgery. Summarized in Additional file 8: Table S4 were the clinicopathological characteristics of the 147 patients, including gender, age, tumor size, CEA, SCCA, histologic grade, smoking status, alcohol consumption, TNM stage and clinical stage. The tumors were staged according to the 7th edition of tumor-node-metastasis (TNM) classification for esophageal carcinoma (UICC, 2009) [43].

One hundred and twenty-three adult healthy volunteer donors were enrolled as a normal control group. None of the donors have any esophageal disease or any other types of malignancy, with information detailed in Additional file 8: Table S4.

Fresh tumor tissues and paired adjacent normal tissues were obtained from ESCC patients and were immediately frozen in liquid nitrogen and then stored at -80°C until RNA extraction.

Peripheral blood from ESCC patients was drawn before and 14d after surgery. Up to 5 ml of blood was collected from each subject in a K_2_EDTA plasma tube and was processed within 1 h for plasma collection. For serum collection, all blood samples were allowed to clot at room temperature for a minimum of 30 min and a maximum of 2 h. Cell and cellular components-free plasma or serum was isolated from all blood samples using a two-step centrifugation protocol (2000 g for 10 min at 4°C, 12000 g for 10 min at 4°C) to thoroughly remove cellular nucleic acids. After separation, plasma and serum samples were transferred to RNase DNase-free tubes and stored at -80°C until total RNA extraction. Blood samples with hemolysis were excluded.

### Cell culture

All cells were a generous gift from Dr. Zhi-Hua Liu, the State Key Laboratory of Molecular Oncology, Cancer Institute, Chinese Academy of Medical Sciences (Beijing, China). Human KYSE30, KYSE70, KYSE450, Eca 109 and HET-1A were cultured in RPMI medium 1640 (Invitrogen, Carlsbad, CA) containing 10% fetal bovine serum (Gibco, Grand Island, NY) and 1% penicillin-streptomycin at 37°C in 5% CO_2_. Cells were plated in 6-well plate at a density of 2 × 10^5^ per well, and then the medium was switched to fresh RPMI-1640 ~ 12 h after plating. After incubation for 3 days, the cells and the cell culture media were separately collected for RNA isolation. The processing of conditioned media was the same as that described for plasma collection.

### Xenograft experiments

The animal study was performed in accordance with the NIH animal use guidelines to explore the source of circulating lncRNAs. In brief, KYSE30 cells were collected at exponential growing stage when they reached ~70% confluence. About 1 × 10^6^ cells in 50% matrigel were injected subcutaneously into the flanks of the BALB/C nude mice (n = 12). An equal number of mice were injected with 100 μl of 50% matrigel in PBS as controls. Four weeks after implantation, mice were anesthetized and their blood was collected in EDTA tube using cardiac puncture and processed for isolation of plasma.

### RNA isolation

RNA extraction from tissues and cultured cells was performed using Trizol reagent (Invitrogen, Carlsbad, CA, USA), whereas total RNA in plasma or cell culture media was isolated by using mirVana PARIS Kit (Ambion 1556, USA). Detailed description of RNA extraction was provided in Supporting Information (Additional file 9: Table S5).

### Quantitative real-time PCR (qPCR)

An aliquot of 1 μg total RNA was reverse transcribed into cDNA using PrimeScript™ RT reagent Kit with gDNA Eraser (Takara: RR047A). The qPCR was then carried out using SYBR® Premix Ex Tag™ II (Takara: RR820A) in 20 μl reactions. All qRT-PCR reactions were performed on ABI 7500 Real-Time PCR System (Applied Biosystems, USA). Each sample was analyzed in triplicate and the specificity of each PCR reaction was confirmed by melt curve analyses. The compliance of the qPCR experiments with the MIQE (Minimum Information for Publication of Quantitative Real-Time PCR Experiments) guidelines was shown in the MIQE checklist [44] (Additional file 9: Table S5). All the qPCR target information were listed in Additional file 10: Table S7.

Four house-keeper genes (*GAPGH*, *TBP*, *β-actin* and *HPRT1*), which have been previously reported to be stably expressed in different human tissues or were once used in ESCC qPCR study, were chosen as candidate endogenous controls for the analysis of tissues and cells lncRNA. RefFinder, a web-based comprehensive tool (http://www.leonxie.com/referencegene.php), was then utilized to evaluate and screen the optimal reference gene.

The expression levels of lncRNAs were calculated using △Ct method, where △Ct = Ct_target_ – Ct_reference_, smaller △Ct value indicates higher expression. Relative expression of lncRNAs was calculated using 2^–△△Ct^ method normalized to endogenous control, with △Ct = Ct_target_ – Ct_reference_, -△△Ct = - (sample △Ct – control △Ct).

All the primers used in the present study were listed in the supporting information (Additional file 11: Table S6).

### Sequencing of qPCR products

After gel extraction and purification, the qPCR products were then cloned into the pUCm-T vector following the manufacturer’s protocol (Sangon Biotech, Shanghai, China), and then sequencing was performed by the Sangon Biotech Co., Ltd.

## Authors’ information

Yu-suo Tong, Xiao-Wei Wang and Xi-lei Zhou considered as joint first authors.

## Electronic supplementary material

Additional file 1: Table S1: Raw Ct values of 21 pairs of ESCC tumor tissues and adjacent normal tissues. (DOC 32 KB)

Additional file 2: Table S2: Correlation between *GAPDH* level (raw Ct value) in human plasma and clinicopathological factors of normal controls and ESCC patients. (DOC 32 KB)

Additional file 3: Text S1: Supplementary data. (DOCX 16 KB)

Additional file 4: Figure S1: Sequencing Results of Plasma qPCR Products of *POU3F3* **(A)**, *HNF1A-AS1* **(B)** and *SPRY4-IT1* **(C)**. (TIFF 3 MB)

Additional file 5: Figure S2: Comparison plasma level of *ENST00000435885* and *AFAP1-AS1* between ESCC and normal control groups. *ENST00000435885* (A) and *AFAP1-AS1* (B) expression showed no significant differences between ESCC and normal controls. Data presented as △Ct values normalized to *GAPDH*. (TIFF 0 bytes)

Additional file 6: Figure S3: Effect of delayed processing of blood on ESCC-related lncRNAs expression. ●, unfiltered plasma incubated at 4°C, △, filtered plasma incubated at room temperature. The symbols represented the means at specified time points. (TIFF 3 MB)

Additional file 7: Table S3: Correlation between ESCC-related lncRNAs expression (△Ct) in plasma and clinicopathological characteristics of 143 ESCC patients. (DOC 64 KB)

Additional file 8: Table S4: Clinical characteristics of study population. (DOC 54 KB)

Additional file 9: Table S5: Checklist MIQE. (DOC 116 KB)

Additional file 10: Table S7: qPCR target information. (DOC 54 KB)

Additional file 11: Table S6: Primers sequences. (DOC 116 KB)

## Abbreviations

lncRNA: Long non-coding RNA
ESCC: Esophageal squamous cell carcinoma
ROC: Receiver operating characteristic curve
AUC: Area under the ROC curve
SCCA: Squamous cell carcinoma antigen
CEA: Carcinoembryonic antigen
CA 19–9: Carbohydrate antigen (CA) 19–9
TNM: Tumor-node-metastasis
q-PCR: Quantitative real-time polymerase chain reaction
CI: Confidence interval.
