# Supplementary material for: Identification of the long non-coding RNA POU3F3 in plasma as a novel biomarker for diagnosis of esophageal squamous cell carcinoma
Source: Mol Cancer. 2015 Jan 21;14:3. doi: 10.1186/1476-4598-14-3 (PMC4631113; doi:10.1186/1476-4598-14-3)
Supplement: Supplementary file 1 — Additional file 1: Table S1: Raw Ct values of 21 pairs of ESCC tumor tissues and adjacent normal tissues. (DOC 32 KB) [file 12943_2014_1498_MOESM1_ESM.doc]

**Table S2** Correlation between *GAPDH* level (raw Ct value) in human plasma and clinicopathological factors of normal controls and ESCC patients

| Variable | No. of patients (%) | *GAPDH* level  (Mean ± SD) | *p*-value |
| --- | --- | --- | --- |
| Age (years) |  |  |  |
| <55 | 42 | 30.245 ± 2.084 | 0.952 |
| ≥55 | 228 | 30.293 ± 2.303 |  |
| Gender |  |  |  |
| Male | 170 | 30.480 ± 2.398 | 0.233 |
| Female | 100 | 29.953 ± 1.992 |  |
| Pathological diagnosis |  |  |  |
| ESCC | 147 | 30.166 ± 2.326 | 0.277 |
| Healthy controls | 123 | 30.427 ± 2.194 |  |
