# Supplementary material for: Identification of the long non-coding RNA POU3F3 in plasma as a novel biomarker for diagnosis of esophageal squamous cell carcinoma
Source: Mol Cancer. 2015 Jan 21;14:3. doi: 10.1186/1476-4598-14-3 (PMC4631113; doi:10.1186/1476-4598-14-3)
Supplement: Supplementary file 3 — Additional file 3: Text S1: Supplementary data. (DOCX 16 KB) [file 12943_2014_1498_MOESM3_ESM.docx]

Additional file 3: Text S1 Supplementary data.

***Optimisation of plasma RNA isolation techniques.*** Prerequisite to developing circulating lncRNAs-based tumor markers is the ability to detect lncRNAs from plasma or serum with sufficient sensitivity. Therefore, optimization of extraction method was very important. Three extraction methods (Trizol LS reagent, mirVana PARIS Kit, and Trizol reagent) which have been reported to isolate total RNA from blood samples were then selected to compare their extraction efficiency. Four healthy participants were used in this pilot experiment, and each plasma sample was divided into three parts for three different extraction methods. The yield and quality of total RNA was evaluated by measuring the absorbance at 260 and 280 nm. Although these three approaches have been proven effective, mirVana PARIS Kit approach was chosen because of the highest RNA yield and quality.
