# Supplementary figures and images for: Identification of the long non-coding RNA POU3F3 in plasma as a novel biomarker for diagnosis of esophageal squamous cell carcinoma
Source: Mol Cancer. 2015 Jan 21;14:3. doi: 10.1186/1476-4598-14-3 (PMC4631113; doi:10.1186/1476-4598-14-3)

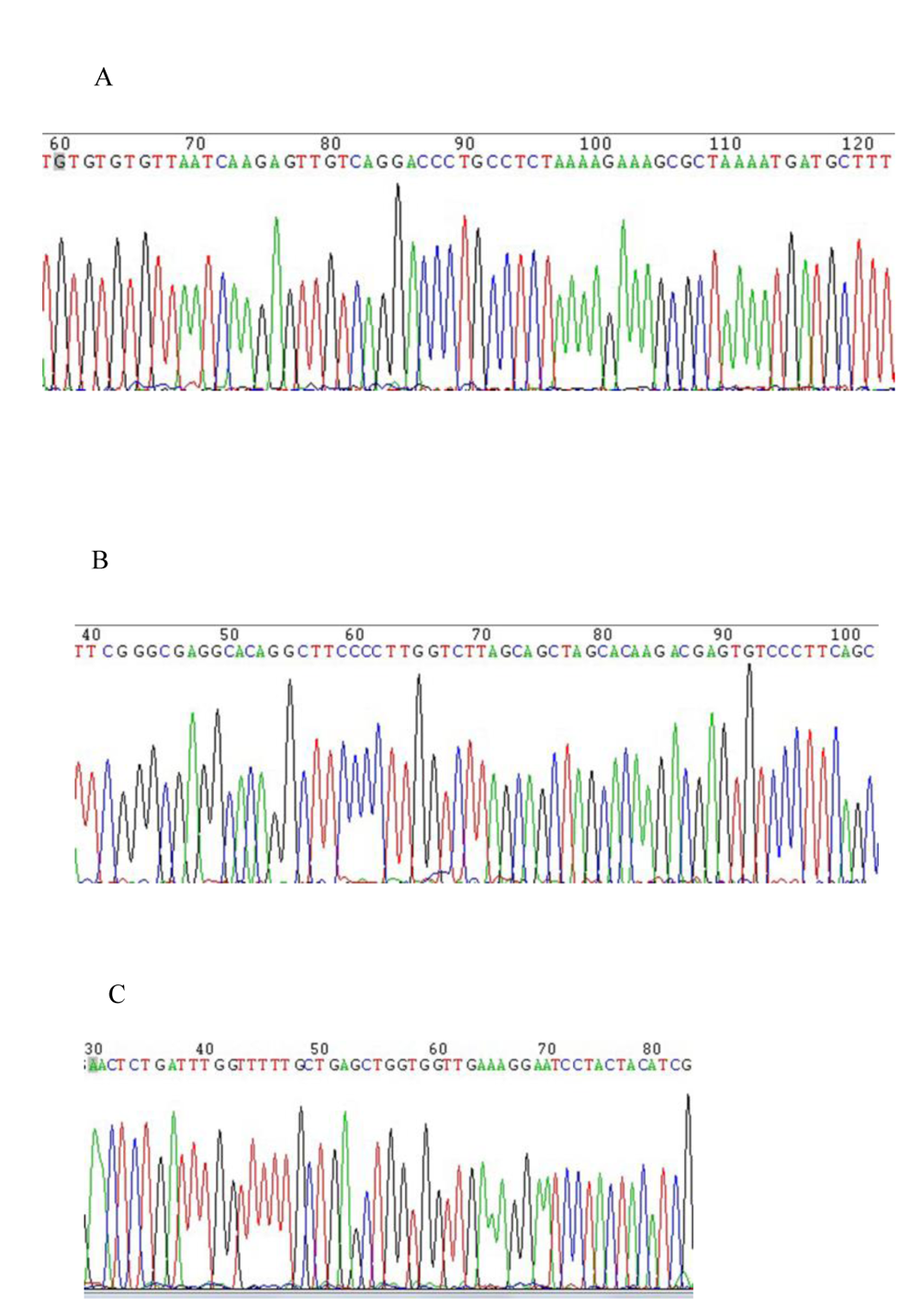

Supplement: Supplementary file 4 — Additional file 4: Figure S1: Sequencing Results of Plasma qPCR Products of POU3F3 (A), HNF1A-AS1 (B) and SPRY4-IT1 (C). (TIFF 3 MB) [file 12943_2014_1498_MOESM4_ESM.tiff]

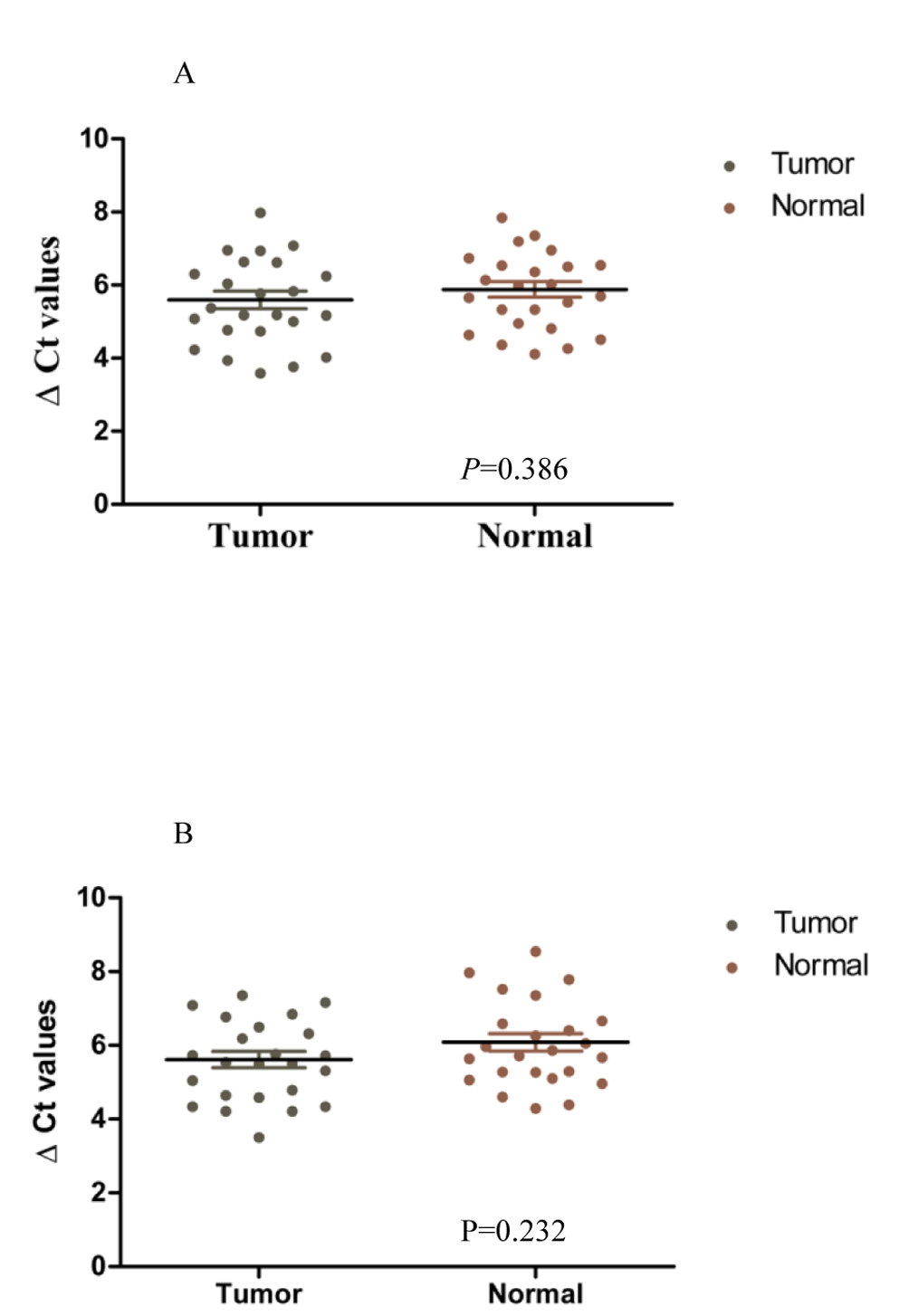

Supplement: Supplementary file 5 — Additional file 5: Figure S2: Comparison plasma level of ENST00000435885 and AFAP1-AS1 between ESCC and normal control groups. ENST00000435885 (A) and AFAP1-AS1 (B) expression showed no significant differences between ESCC and normal controls. Data presented as △Ct values normalized to GAPDH. (TIFF 0 bytes) [file 12943_2014_1498_MOESM5_ESM.tiff]

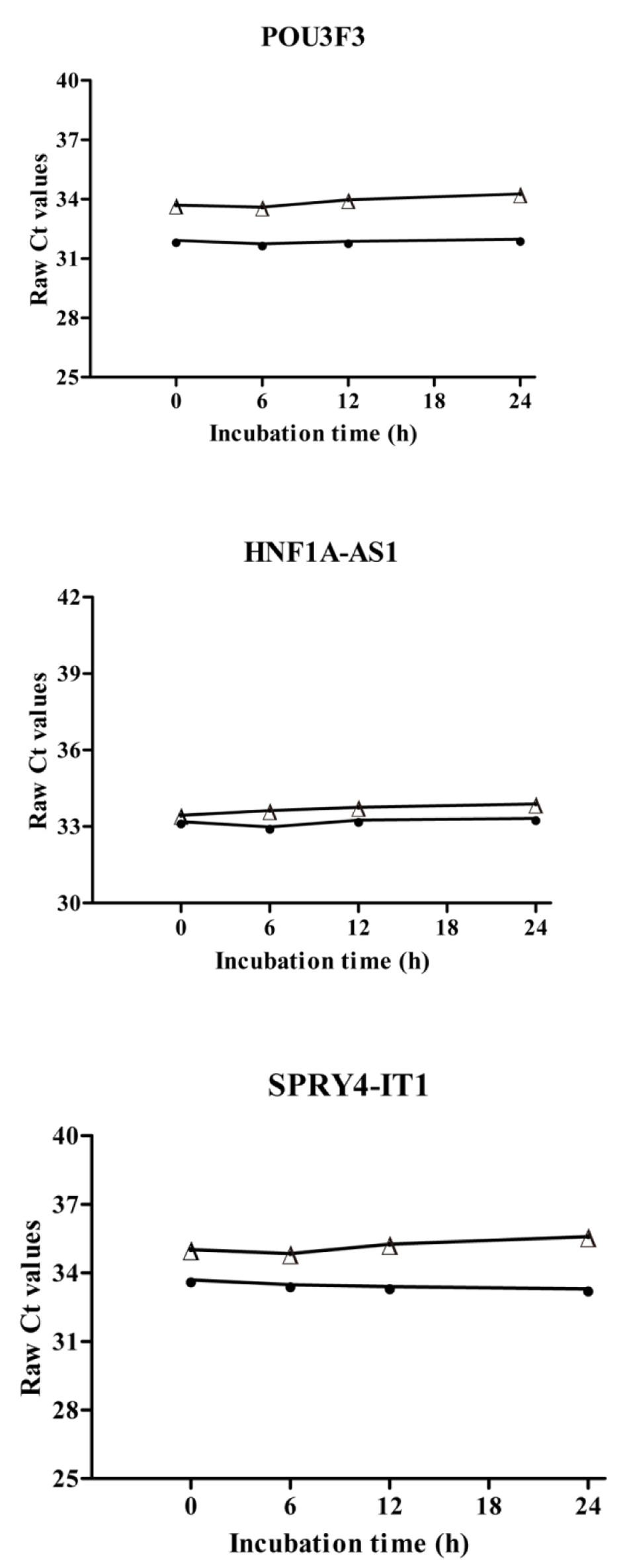

Supplement: Supplementary file 6 — Additional file 6: Figure S3: Effect of delayed processing of blood on ESCC-related lncRNAs expression. ●, unfiltered plasma incubated at 4°C, △, filtered plasma incubated at room temperature. The symbols represented the means at specified time points. (TIFF 3 MB) [file 12943_2014_1498_MOESM6_ESM.tiff]
