# Supplementary material for: Identification of the long non-coding RNA POU3F3 in plasma as a novel biomarker for diagnosis of esophageal squamous cell carcinoma
Source: Mol Cancer. 2015 Jan 21;14:3. doi: 10.1186/1476-4598-14-3 (PMC4631113; doi:10.1186/1476-4598-14-3)
Supplement: Supplementary file 7 — Additional file 7: Table S3: Correlation between ESCC-related lncRNAs expression (△Ct) in plasma and clinicopathological characteristics of 143 ESCC patients. (DOC 64 KB) [file 12943_2014_1498_MOESM7_ESM.doc]

**Table S3** Correlation between ESCC-related lncRNAs expression (△Ct) in plasma and clinicopathological characteristics of 143 ESCC patients

| Variables | No. of case (%) | *POU3F3*  Level: Mean ± SD | *p*-value | *HNF1A-AS1*  Level: Mean ± SD | *p*-value | *SPRY4-IT1*  Level: Mean ± SD | *p*-value |
| --- | --- | --- | --- | --- | --- | --- | --- |
| Age |  |  | 0.171 |  | 0.377 |  | 0.725 |
| <55 | 18 (12.2%) | 5.039 ± 1.171 |  | 5.395 ± 1.478 |  | 5.524 ± 1.263 |  |
| ≥55 | 129 (87.8%) | 5.488 ± 1.313 |  | 5.748 ± 1.598 |  | 5.639 ± 1.309 |  |
| Gender |  |  | 0.236 |  | 0.541 |  |  |
| Male | 98 (67%) | 5.343 ± 1.234 |  | 5.762 ± 1.670 |  | 5.722 ± 1.296 | 0.201 |
| Female | 49 (33%) | 5.614 ± 1.424 |  | 5.592 ± 1.402 |  | 5.431 ± 1.300 |  |
| Smoking status |  |  | 0.289 |  | 0.980 |  | 0.666 |
| Ever or current | 56 (38%) | 5.289 ± 1.154 |  | 5.700 ± 1.282 |  | 5.662 ± 1.380 |  |
| Never | 91 (62%) | 5.524 ± 1.384 |  | 5.708 ± 1.759 |  | 5.566 ± 1.168 |  |
| Alcohol consumption |  |  | 0.883 |  | 0.775 |  | 0.272 |
| Ever or current | 44 (30%) | 5.444 ± 1.291 |  | 5.763± 1.168 |  | 5.444 ± 1.368 |  |
| Never | 103 (70%) | 5.410 ± 1.341 |  | 5.681 ± 1.735 |  | 5.701 ± 1.119 |  |
| Location |  |  | 0.164 |  | 0.598 |  | 0.419 |
| Cervical/upper thoracic | 13 (9%) | 5.914 ± 1.234 |  | 5.927 ± 1.201 |  | 5.905 ± 0.909 |  |
| Middle/lower thoracic | 134 (91%) | 5.387 ± 1.303 |  | 5.683 ± 1.618 |  | 5.598 ± 1.331 |  |
| Tumor size |  |  | 0.644 |  | 0.260 |  | 0.604 |
| ≤5cm | 99 (67%) | 5.469 ± 1.308 |  | 5.602 ± 1.256 |  | 5.586 ± 1.278 |  |
| >5cm | 48 (33%) | 5.362 ± 1.300 |  | 5.917 ± 2.104 |  | 5.705 ± 1.355 |  |
| Histological grade |  |  | 0.107 |  | 0.257 |  | 0.840 |
| G1 | 15 (10%) | 6.106 ± 1.103 |  | 6.345 ± 3.232 |  | 5.783 ± 1.013 |  |
| G2 | 88 (60%) | 5.351 ± 1.287 |  | 5.626 ± 1.399 |  | 5.633 ± 1.279 |  |
| G3/G4 | 44 (30%) | 5.371 ± 1.353 |  | 5.645 ± 1.149 |  | 5.555 ± 1.443 |  |
| T status |  |  | 0.977 |  | 0.183 |  | 0.092 |
| T1,T2 | 36 (24%) | 5.440 ± 1.388 |  | 5.399 ± 1.228 |  | 5.308 ± 1.359 |  |
| T3,T4 | 111 (76%) | 5.442 ± 1.280 |  | 5.805 ± 1.675 |  | 5.728 ± 1.270 |  |
| N status |  |  | 0.741 |  | 0.175 |  | 0.069 |
| N0 | 59 (40%) | 5.390 ± 1.325 |  | 5.488 ± 1.292 |  | 5.387 ± 1.313 |  |
| N1 | 88 (60%) | 5.463 ± 1.293 |  | 5.851 ± 1.744 |  | 5.785 ± 1.214 |  |
| M status |  |  | 0.457 |  | 0.462 |  | 0.619 |
| M0 | 116 (78.9%) | 5.392 ± 1.271 |  | 5.655 ± 1.267 |  | 5.653 ± 1.319 |  |
| M1-lym | 31 (21.1%) | 5.589 ±1.421 |  | 5.892 ± 2.450 |  | 5.521 ±1.241 |  |
| Clinical stage |  |  | 0.826 |  | 0.260 |  | 0.131 |
| I and II | 65 (44%) | 5.407 ± 1.293 |  | 5.539 ± 1.326 |  | 5.443 ± 1.355 |  |
| III and IV | 82 (56%) | 5.455 ± 1.317 |  | 5.837 ± 1.758 |  | 5.770 ± 1.244 |  |

M1-lym, distant lymph node metastasis
