# Supplementary material for: Identification of the long non-coding RNA POU3F3 in plasma as a novel biomarker for diagnosis of esophageal squamous cell carcinoma
Source: Mol Cancer. 2015 Jan 21;14:3. doi: 10.1186/1476-4598-14-3 (PMC4631113; doi:10.1186/1476-4598-14-3)
Supplement: Supplementary file 8 — Additional file 8: Table S4: Clinical characteristics of study population. (DOC 54 KB) [file 12943_2014_1498_MOESM8_ESM.doc]

**Table S4** Clinical characteristics of study population

| Characteristic | Patients (n=147) | Controls  (n=123) | *p*-value |
| --- | --- | --- | --- |
| Age |  |  | 0.129 |
| ≤55 | 18 (12.2%) | 24 (19.5%) |  |
| >55 | 129 (87.8%) | 99 (80.5%) |  |
| Gender |  |  | 0.206 |
| Male | 98 (67%) | 72 (58.5%) |  |
| Female | 49 (33%) | 51 (41.5%) |  |
| Smoking status |  |  | 0.199 |
| Ever or current | 56 (38%) | 37 (30.1%) |  |
| Never | 91 (62%) | 86 (69.9%) |  |
| Alcohol consumption |  |  | 0.306 |
| Ever or current | 44 (30%) | 31 (25.2%) |  |
| Never | 103 (70%) | 92 (74.8%) |  |
| Tumor location |  |  | NA |
| Cervical/upper thoracic | 13 (9%) |  |  |
| Middle/lower thoracic | 134 (91%) |  |  |
| Tumor size |  |  | NA |
| ≤5cm | 99 (67%) |  |  |
| >5cm | 48 (33%) |  |  |
| Histological grade |  |  | NA |
| G1 | 15 (10%) |  |  |
| G2 | 88 (60%) |  |  |
| G3/G4 | 44 (30%) |  |  |
| Depth of invasion |  |  | NA |
| T1,T2 | 36 (24%) |  |  |
| T3,T4 | 111 (76%) |  |  |
| Lymph node metastasis |  |  | NA |
| Negative | 59 (40%) |  |  |
| Positive | 88 (60%) |  |  |
| Clinical stage |  |  | NA |
| I | 26 (18%) |  |  |
| II | 39 (26%) |  |  |
| III | 51 (35%) |  |  |
| IV | 31 (21%) |  |  |
| CEA |  |  | <0.001 |
| Negative | 99 (67.3%) | 120 (97.6%) |  |
| Positive | 48 (32.7%) | 3 (3.4%) |  |
| SCCA |  |  | <0.001 |
| Negative | 60 (40.8%) | 115 (93.5%) |  |
| Positive | 87 (59.2%) | 8 (6.5%) |  |

Abbreviations: *CEA*, carcinoembryonic antigen, *SCCA*, squamous cell carcinoma antigen. The cut-off values for *CEA* and *SCCA* were 5ng/ml and 1.5ng/ml, respectively. Statistic analysis, Pearson Chi-Square.
