# Supplementary material for: Identification of the long non-coding RNA POU3F3 in plasma as a novel biomarker for diagnosis of esophageal squamous cell carcinoma
Source: Mol Cancer. 2015 Jan 21;14:3. doi: 10.1186/1476-4598-14-3 (PMC4631113; doi:10.1186/1476-4598-14-3)
Supplement: Supplementary file 9 — Additional file 9: Table S5: Checklist MIQE. (DOC 116 KB) [file 12943_2014_1498_MOESM9_ESM.doc]

Table S5 checklist MIQE

Item to check Importance

|  |  | **Experimental design** |
| --- | --- | --- |
| Definition of experimental and control groups | E | **Experimental groups**: patients who were newly diagnosed with ESCC. **Control groups**: adult healthy volunteer donors |
| Number within each group | E | n=147 for experimental group, and n=123 for normal control group |
| Assay carried out by the core or investigator’s laboratory? | D |  |
| Acknowledgment of authors’ contributions | D |  |
|  |  | **Sample** |
| Description | E | Tumor tissues and paired adjacent normal samples were obtained from ESCC patients. Blood samples were drawn from each participant and peripheral blood from ESCC patients was drawn before and 14d after surgery. |
| Volume/mass of sample processed | D | For tissue samples, 40-60mg. For blood samples, 5ml. |
| Microdissection or macrodissection | E | Macrodissection. |
| Processing procedure | E | All blood samples were subjected to isolation of cell-free nucleic acids using a two-step centrifugation protocol (2000g for 10 min at 4°C, 12000g for 10 min at 4°C). |
| If frozen, how and how quickly? | E | Tumor tissues and paired adjacent normal samples were immediately frozen in liquid nitrogen after they were washed with pre-cold PBS. |
| If fixed, with what and how quickly? | E | Not fixed. |
| Sample storage conditions and duration | E | Samples were stored at –80°C until RNA extraction. |
|  |  | **Nucleic acid extraction** |
| Procedure and/or instruments | E | ***RNA extraction from tissues and cultured cells.*** Total RNAs were extracted from tumor tissues and cultured cells using Trizol reagent according to the manufacturer’s instructions. Briefly, 1 ml of Trizol Reagent was added to 50-100 mg of tissue. For cells RNA extraction, cells were washed twice with iced PBS. One milliliter of Trizol Reagent was added directly to the culture dish to lyse the cells. Lysates were harvested manually with passing the cell lysate several times through a pipette and transferred to a 1.5ml RNase DNase-free tube. Vortexed the homogenized samples well and then incubated for 5 min at room temperature to completely dissociate nucleoprotein complexes. After homogenization, phase separation was performed with 200μl of chloroform at 12000g for 15min. Then carefully transferred the aqueous phase to a fresh tube, avoiding the interphase altogether. After supplying 500μl of isopropyl alcohol, the mixture was allowed to incubate at room temperature for 10 min. Following centrifugation at 12000g for 10 min total RNA was precipitated from the aqueous phase. Continue with the manufacturer’s protocol as described. After washing once with 1ml of 75% ethanol, centrifugation was done at 7500g for 5 min at 4°C. Finally, RNA pellet was resuspended in 40μl RNase-free water and stored at –80°C. ***RNA extraction from plasma, serum and cell culture media****.*Isolation of lncRNAs from liquid samples was performed using mirVana PARIS Kit. In brief, all plasma or serum samples were thawed on ice, and 400μl of each sample was transferred to a tube containing with an equal volume of 2×Denaturing Solution. Then added a volume of Acid-Phenol: Chloroform equal to the total volume of the sample lysate plus the 2X Denaturing Solution, shaking for 45 sec to completely mix. Following the centrifugation at 10000g for 5min total RNA was precipitated from the aqueous phase by adding 1.25 volumes 100% ethanol. Subsequently, pass the lysate/ethanol mixture through a Filter Cartridge, and wash the filter with wash solution. From the columns, RNA was eluted in 50μl of RNase-free water. The average volume of eluate recovered from each column was 45μl. |
| Name of kit and details of any modifications | E | Trizol reagent (Invitrogen, Carlsbad, CA, USA), mirVana PARIS Kit (Ambion 1556, USA). We exactly followed the manufacture’s protocol |
| Source of addition reagents used | D | Chloroform (Merck); isopropyl alcohol (Beyotime); Ethanol (Beyotime); DEPC-Treated Water (Ambion) |
| Details of DNase or RNase treatment | E | 1ug of RNA was treated with 1μl of gDNA Erase and 2μl 5× gDNA Eraser Buffer in a 20ul final volume reaction. Digestion of DNA was achieved with2 minutes incubation at 42 °C. |
| Contamination assessment (DNA or RNA) | E | Reverse transcription controls (without enzyme) were performed in order to assess the existence of DNA in the RNA sample. For that purpose, RNA was processed as a normal sample in the RT step, except that no reverse transcriptase was added to the reaction mixture. |
| Nuclei acid quantification | E | RNA concentration was assessed using spectrophotometry by measuring the absorbance at 260 nm UV light. |
| Instrument and method | E | Spectrophotometry (Eppendorf) |
| Purity (A260/A280) | D | RNA purity was determined by measuring the absorbance at OD260 and OD280. |
| Yield | D | RNA yield was calculated using spectrophotometry. The average yield was about 1200ng/μl for tissue RNA and approximate 190ng/μl for plasma RNA. |
| RNA integrity: method/instrument | E | RNA integrity was assessed using Agilent’s 2100 bioanalyzer (Agilent Technologies, Santa Clara, CA, USA). |
| RIN/RQI or Cq of 3' and 5' transcripts | E | Not applicable |
| Electrophoresis traces | D |  |
| Inhibition testing (Cq dilutions, spike, or other) | E | The standard curve has been considered sufficient to rule out the presence of inhibitors of reverse-transcription activity or PCR also taking into account the high quality of starting RNAs. |
|  |  | **Reverse transcription** |
| Complete reaction conditions | E | Reverse transcription was carried out in 20μl reaction volume. Briefly, 1μg total RNA, 2μl 5× gDNA Eraser Buffer, 1μl gDNA Eraser and nuclease-free water were mixed well and incubated for 2 min at 42°C to eliminate genomic DNA. For the synthesis of cDNA, 4μl 5× PrimeScript Buffer 2, 1μl PrimeScript RT Enzyme Mix 1, 1μl RT Primer Mix and 4μl nuclease-Free water were then added, and the reaction mixtures were performed using the following conditions: 37°C for 15 min, followed by 85°C 5s and then held at 4°C. |
| Amount of RNA and reaction volume | E | Amount of RNA: 1000ng; Reaction volume: 20ul |
| Priming oligonucleotide (if using GSP) and concentration | E | Oligo dTprimer: 50pmol/20μl reaction system |
| Reverse transcriptase and concentration | E | PrimeScript RT Enzyme Mix 1 |
| Temperature and time | E | Specified in "Complete reaction conditions" |
| Manufacturer of reagents and catalogue numbers | D | PrimeScript™ RT reagent kit with gDNA Eraser (Takara: RR047A) |
| Cqs with and without reverse transcription | D |  |
| Storage conditions of cDNA | D | -20°C |
|  |  | qPCR target information |
| Gene symbol | E | Table S7 in supporting information |
| Sequence accession number | E | Table S7 in supporting information |
| Location of amplicon | D |  |
| Amplicon length | E | Table S7 in supporting information |
| In silico specificity screen (Blast, and so on) | E | Described in text |
| Pseudogenes, retropseudogenes, or other homologs? | D |  |
| Sequence alignment | D |  |
| Secondary structure analysis of amplicon | D |  |
| Location of each primer by exon or intron (if applicable) | E | Not applicable. |
| What splice variants are targeted? | E |  |
|  |  | qPCR oligonucleotides |
| Primers sequences | E | Table S6 in supporting information |
| RTPrimerDB identification number | D |  |
| Probe sequences | D |  |
| Location and identity of any modifications | E | No modifications were done. |
| Manufacturer of oligonucleotides | D | Invitrogen |
| Purification method | D | PAGE method |
|  |  | qPCR protocol |
| Complete reaction conditions | E | qPCR was carried out using SYBR® Premix Ex Tag^TM^ Ⅱ (Takara: RR820A) in 20μl reaction volumes. In brief, each reaction was comprised of 2μl of the cDNA solution, 10μl of SYBR® Premix Ex Tag^TM^ (2×), 1.6μl of primers, 0.4μl of ROX Reference Dye Ⅱ and 6μl of nuclease-free water. All qPCR reactions were performed on ABI 7500 Real-Time PCR System (Applied Biosystems, USA), using the following conditions: 95°C for 30 s, followed by 40 cycles at 95°C for 5 s and 60°C for 34s. |
| Reaction volume and amount of cDNA/DNA | E | Reaction volume: 20ul; amount of cDNA: 2ul. |
| Primer, (probe), Mg2, and dNTP concentrations | E | 0.4μM Primers; 2 mM MgCl2; 0.2 mM dNTP |
| Polymerase identity and concentration | E | TakaRa Ex Taq HS |
| Buffer/kit identity and manufacturer | E | TakaRa |
| Exact chemical composition of the buffer | D |  |
| Additives (SYBR Green I, DMSO, and so forth) | E | SYBR Green I |
| Manufacturer of plates/tubes and catalog number | D | PCR Strip Tubes (PCR-0208-C) and PCR Strip Caps (PCR-2CP-RT-C), both provided by Axygen. |
| Complete thermocycling parameters | E | Initial denaturation: 95°C for 30 s, followed by 40 cycles at 95°C for 5 s and 60°C for 34s. |
| Reaction setup (manual/robotic) | D | Manual |
| Manufacturer of qPCR instrument | E | ABI 7500 Real-Time PCR System (Applied Biosystems, USA) |
|  |  | qPCR validation |
| Evidence of optimization (from gradients) | D |  |
| Specificity (gel, sequence, melt, or digest) | E | Specificity was confirmed by melt curve analyses and sequencing analysis (which was provided in Supplementary data). Gene-specific amplification was confirmed by a single band in 2% agarose gel electrophoresis stained with ethidium bromide. No template controls were run for each gene to detect unspecific amplification and primer dimerization. |
| For SYBR Green I, Cq of the NTC | E | The signal of the amplification plot was very late (Cq>38) and therefore there was a high Cq value difference between the negative control and all the cDNA samples. |
| Calibration curves with slope and y intercept | E | *GAPDH*: y = -3.4779x + 24.184; *TBP*: y = -3.4748x + 35.735; *HPRT1*: y = -3.4624x + 34.76; *β-actin*: y = -3.5177x + 33.337; *HOTAIR*: y = -3.4218x + 36.735; *AFAP1-AS1*: y = -3.2956x + 35.884; *HNF1A-AS1*: y = -3.4314x + 36.56; *SPRY4-IT1*: y = -3.3409x + 35.767; *POU3F3*: -3.4608x + 33.465; *91H*: y = - 3.4182x + 36.923; *PlncRNA1*: y = -3.51x + 35.098; *ENST00000435885.1*: y = -3.4412x + 34.503; *XLOC_013104*: y = -3.5538x + 34.008;  *ENST00000547963.1*: y = - 3.4248 + 35.317. |
| PCR efficiency calculated from slope | E | *GAPDH*: 93.9%; *TBP*: 93.9%; *HPRT1*: 94.4%; *β-actin*: 92.4%; *HOTAIR*: 95.9%; *AFAP1-AS1*: 100.1%; *HNF1A-AS1*: 95.6%; *SPRY4-IT1*: 99.2%; *POU3F3*: 94.5%; *91H*: 96.1%; *PlncRNA1*: 92.7%; *ENST00000435885.1*: 95.2%; *XLOC_013104*: 91.2%; *ENST00000547963.1*: 95.9 %. |
| CIs for PCR efficiency or SE | D |  |
| r2 of calibration curve | E | *GAPDH*: 0.9981; *TBP*: 0.9830; *HPRT1*: 0.9794; *β-actin*: 0.9930; *HOTAIR*: 0.9970; *AFAP1-AS1*: 0.9867; *HNF1A-AS1*: 0.9850; *SPRY4-IT1*: 0.9910; *POU3F3*: 0.9952; *91H*: 0.9870; *PlncRNA1*: 0.9920; *ENST00000435885.1*: 0.9790; *XLOC_013104*: 0.9934; *ENST00000547963.1*: 0.9860. |
| Linear dynamic range | E | In average, linear dynamic range was considered taking into account the linearity of the standard curves; from 1/10 dilution of cDNA to 1/1000 dilution. |
| Cq variation at LOD | E |  |
| CIs throughout range | D |  |
| Evidence for LOD | D |  |
| If multiplex, efficiency and LOD of each assay | E | Not applicable |
|  |  | Data analysis |
| qPCR analysis program (source, version) | E | Quantification Software version 2.1 (Applied Biosystems, USA) |
| Method of Cq determination | E | The threshold was defined as the fractional cycle number at which the fluorescence exceeded the given threshold and was calculated using SDS Relative Quantification Software version 2.1 using the automatic baseline setting. |
| Outlier identification and disposition | E | lncRNAs with the Cq values greater than 40 were defined the Cq values of 40 |
| Results for NTCs | E | The signal of the amplification plot was very late (Cq>38) and therefore there was a high Cq value difference between the negative control and all the cDNA samples. |
| Justification of number and choice of reference genes | E | RefFinder, a web-based comprehensive tool, was then utilized to evaluate and screen the optimal reference gene for tissue lncRNAs analysis. Statistical analysis was performed to investigate the optimal reference gene for plasma lncRNAs analysis. |
| Description of normalization method | E | Described in text. |
| Number and concordance of biological replicates | D |  |
| Number and stage (reverse transcription or qPCR) of technical replicates | E | qPCR reactions were performed in triplicate. |
| Repeatability (intraassay variation) | E | Mean standard deviation of triplicates: less than 0.10. |
| Reproducibility (interassay variation, CV) | D |  |
| Power analysis | D |  |
| Statistical methods for results significance | E | The statistical significance of tissue, cell, plasma, and serum lncRNA levels was analyzed by Mann-Whitney, Wilcoxon, χ^2^, and Kruskal-Wallis tests where appropriate. |
| Software (source, version) | E | SPSS software (version 20.0) |
| Cq or raw data submission with RDML | D |  |
